# Calculation and plotting of retinal nerve fiber paths based on Jansonius et al. 2009/2012 with an R program

**DOI:** 10.1016/j.dib.2018.02.065

**Published:** 2018-02-28

**Authors:** M. Bach, M.B. Hoffmann

**Affiliations:** a University Eye Center, Medical Center – University of Freiburg, Germany; b Faculty of Medicine, University of Freiburg, Germany; c Visual Processing Laboratory, Universitäts-Augenklinik, Magdeburg, Germany; d Center for Behavioural Brain Sciences, Magdeburg, Germany

## Abstract

The data presented in this article are related to the research article entitled “Retinal conduction speed analysis reveals different origins of the P50 and N95 components of the (multifocal) pattern electroretinogram” (Bach et al., 2018) [1]. That analysis required the individual length data of the retinal nerve fibers (from ganglion cell body to optic nerve head, depending on the position of the ganglion cell body). Jansonius et al. (2009, 2012) [2,3] mathematically modeled the path morphology of the human retinal nerve fibers. We here present a working implementation with source code (for the free and open-source programming environment “R”) of the Jansonius’ formulas, including all errata. One file defines Jansonius et al.’s “phi” function. This function allows quantitative modelling of paths (and any measures derived from them) of the retinal nerve fibers. As a working demonstration, a second file contains a graph which plots samples of nerve fibers. The included R code runs in base R without the need of any additional packages.

**Specifications Table**

**Table:** 

Subject area	*neuroscience, ophthalmology, computational vision*
More specific subject area	*retinal morphology*
Type of data	*R source code and graph*
How data was acquired	*prior published functions were amended with errata implemented in R, and debugged*
Data format	*R source code as plain text file including all pertinent data constants*
Experimental factors	*none*
Experimental features	*allows quantitative modeling of retinal nerve fibers*
Data source location	*attached*
Data accessibility	*data accompanies this article: 2 plain text files “phiFunction-definition.R” and “phiFunction-demoPlot.R”*

**Value of the data**

• The published function to model retinal nerve fibers initially contained errors and spreads over errata.
• The programming environment R [4] is open-source and available free at http://www.r-project.org, the present function works in a basic R installation without additional packages.
• This working phi function allows quantitative modelling of paths and any derivative measures of the retinal nerve fibers.
• We include a working demonstration with a plot of nerve fiber paths.

## 1 Data

The data consists of 2 plain text files “phiFunction-definition.R” and “phiFunction-demoPlot.R” which are ready to be executed in a plain R [4] environment. All data is expressed in the program code and appropriate constants.

## 2 Experimental design, materials and methods

**Fig. 1:**
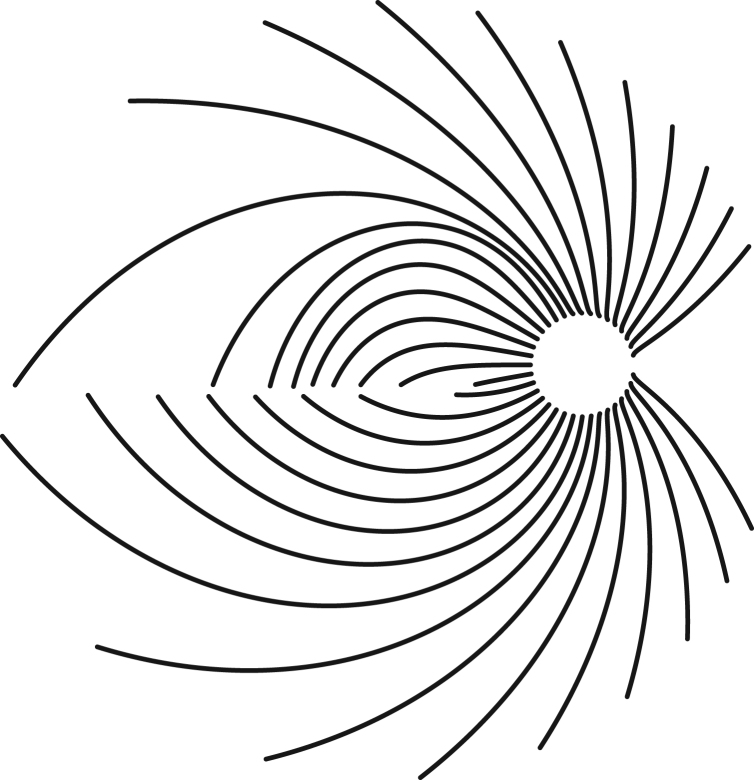
A schematic view of the retina, ±30° of eccentricity. From the optic nerve head (spared circular area on the right) the fibers depart, calculated based on Jansonius et al. [2], [3] as implemented in the R source code here.

With a standard installation of “R”, open and execute “phiFunction-definition.R”. Then execute “phiFunction-demoPlot.R”. A graph containing a set of fiber bundle traces is created, thus demonstrating the validity of the phi function and recreating the accompanying Fig. 1. The program was the basis to assess conduction times from retinal ganglion cell bodies to the optic nerve head [1].
